# Erythrocyte Catalase Activity in More Frequent Microcytic Hypochromic Anemia: Beta-Thalassemia Trait and Iron Deficiency Anemia

**DOI:** 10.1155/2015/343571

**Published:** 2015-10-07

**Authors:** Sandra Stella Lazarte, María Eugenia Mónaco, Cecilia Laura Jimenez, Miryam Emilse Ledesma Achem, Magdalena María Terán, Blanca Alicia Issé

**Affiliations:** ^1^Instituto de Bioquímica Aplicada, Facultad de Bioquímica, Química y Farmacia, Universidad Nacional de Tucumán (UNT), Balcarce 747, San Miguel de Tucumán, 4000 Tucumán, Argentina; ^2^Instituto de Biología, Facultad de Bioquímica, Química y Farmacia, Universidad Nacional de Tucumán, Chacabuco 461, San Miguel de Tucumán, 4000 Tucumán, Argentina

## Abstract

Most common microcytic hypochromic anemias are iron deficiency anemia (IDA) and *β*-thalassemia trait (BTT), in which oxidative stress (OxS) has an essential role. Catalase causes detoxification of H_2_O_2_ in cells, and it is an indispensable antioxidant enzyme. The study was designed to measure erythrocyte catalase activity (ECAT) in patients with IDA (10) or BTT (21), to relate it with thalassemia mutation type (*β* ^0^ or *β* ^+^) and to compare it with normal subjects (67). Ninety-eight individuals were analyzed since September 2013 to June 2014 in Tucumán, Argentina. Total blood count, hemoglobin electrophoresis at alkaline pH, HbA_2_, catalase, and iron status were performed. *β*-thalassemic mutations were determined by real-time PCR. Normal range for ECAT was 70,0–130,0 MU/L. ECAT was increased in 14% (3/21) of BTT subjects and decreased in 40% (4/10) of those with IDA. No significant difference (*p* = 0,245) was shown between normal and BTT groups, while between IDA and normal groups the difference was proved to be significant (*p* = 0,000). In *β* ^0^ and *β* ^+^ groups, no significant difference (*p* = 0,359) was observed. An altered ECAT was detected in IDA and BTT. These results will help to clarify how the catalase activity works in these anemia types.

## 1. Introduction

Normal erythrocytes are protected against potentially dangerous combination of oxygen and iron (hemichromes and heme associated iron) for extremely efficient endogenous mechanisms, such as superoxide dismutase (SOD), catalase, glutathione peroxidase (GPx), reduced glutathione, and vitamin E. Microcytosis is the physiological consequence of reduced hemoglobin content in the red blood cell (RBC) due to a synthesis defect of globin chains or heme [1]. Experimental data in mice showed that the mitotic events during differentiation are associated with a substantial reduction in the mean corpuscular volume (MCV) [2]. In addition to morphological, biochemical, and metabolic changes, small cell erythrocytes are characterized by shorter survival, being the oxidative damage to RBC membrane, one of the underlying mechanisms responsible [3].

Microcytosis is defined by MCV lower than 80 fL and hypochromia through mean corpuscular hemoglobin lower than 27 pg. Hypochromic microcytic anemia may result from an iron deficiency (iron deficiency anemia), a defect in the globin genes (hemoglobinopathies or thalassemia), a defect in heme synthesis (sideroblastic anemia), or a defect in iron availability and acquisition by erythroblast (anemia of chronic disease).

According to World Health Organization (WHO), the primary cause of anemia is iron deficiency, especially in pregnant women and children [4]. Regarding hemoglobinopathies, at present, about 5% of world population is carrier of a potentially pathological hemoglobin gene. Beta- (*β*-) thalassemia is the most common hemoglobinopathy in the Mediterranean basin, the Middle East, and Asia [5]. Accordingly, in Argentina, *β*-thalassemia is the most common inherited anemia [6–8]. Therefore, the most frequent hypochromic microcytic anemias are iron deficiency anemia and *β*-thalassemia.

There are two molecular forms of *β*-thalassemia, *β* ^+^ thalassemia in which a small amount of *β*-globin chains are detectable and *β* ^0^ thalassemia, in which they are undetectable. Till this day there are over 200 gene mutations capable of producing thalassemia phenotypes [9]. Clinically, *β*-thalassemia is classified as minor (asymptomatic) or major (severe anemia), depending on the mutation responsible for this alteration is present in heterozygous or homozygous state. There is also a moderate clinical features syndrome known as thalassemia intermedia (homozygous or double heterozygous). In *β*-thalassemia, as a result of *β*-chains decrease, a relative alpha- (*α*-) chains excess occurs [10]. Free *α*-chains are unable to form viable tetramers and precipitate in RBC precursors in bone marrow forming inclusion bodies called hemichromes. They are responsible for the large intramedullary destruction of erythroblasts and therefore for ineffective erythropoiesis in *β*-thalassemia [11]. Hemichromes also precipitate in membrane of mature RBC and cause changes in its structure which induce lipid peroxidation and exposure of anionic phospholipids, which together leads to premature clearance by the spleen [12]. In both, erythroid precursors and mature RBCs, free iron resulting from heme denaturation produces damage to lipids membrane, cellular proteins, or DNA. Free iron is toxic because it triggers Fenton reaction in which free radicals are formed, increasing cellular oxidative stress (OxS) due to reactive oxygen species (ROS) production, such as superoxide, hydrogen peroxide (H_2_O_2_), and hydroxyl radicals [13].

Several studies have evaluated the oxidant and antioxidant status in thalassemia major and intermedia [14, 15], and in severe *β*-thalassemia it is difficult to evaluate the role played by the antioxidant enzymes because of the relevant proportion of normal red cells due to multiple transfusions. Furthermore little is known about the oxidative status in *β*-thalassemia trait (BTT) subjects and its relation with different *β*-thalassemia mutations. Such assessment is important due to the large genotypic and phenotypic heterogeneity of different populations.

The antioxidant system has been proposed as a biomarker of OxS through measuring detoxifying enzymes such as catalase [16]. The enzyme was first discovered by Louis Jacques Thenard in 1818. It is an intracellular enzyme constituted by four polypeptide chains with four heme-porphyrin groups. The human catalase gene (CAT, NCBI Gene ID: 847) is localized on the short arm of chromosome 11 (11p13) NM 001752.3 and NP 001743.1. Catalase is responsible for detoxification of H_2_O_2_ in cells [15]. Decreased activity of catalase may lead to increased H_2_O_2_ concentration and damage of oxidation sensitive tissues that may contribute to the manifestation of various diseases such as diabetes mellitus and anemia [17].

Iron deficiency affects activity of many iron-dependent enzymes (like catalase), and in iron deficiency anemia (IDA) RBCs are more susceptible to oxidation. RBCs from IDA subjects lyse more readily than normal cells on* in vitro* exposure to H_2_O_2_ [18], suggesting some defect in the protection mechanism of iron-deficient RBCs against oxidant damage. Increased hemoglobin autoxidation and subsequent generation of ROS can account for the shorter RBC lifespan and other pathological changes associated with IDA [19]. The literature offers contradictory and limited data on oxidative stress and antioxidant defense in patients with IDA, and increased [20] and decreased catalase activity [21] have been reported.

The present study was designed to measure catalase activity in individuals suffering from some of the most common microcytic hypochromic anemia, that is, IDA or BTT, and to compare it with normal subjects. It was also proposed to relate the type of *β*-thalassemia mutation with the catalase activity.

## 2. Materials and Methods

*Design*. A descriptive cross-sectional study was conducted.

*Subjects*. The sample consisted of 31 patients who attended* Instituto de Bioquímica Aplicada* (Tucumán, Argentina), for the diagnosis of hereditary anemia during the period of September 2013 up to June 2014. Sixty-seven normal individuals, whose participation was voluntary, were also included. Blood was drawn and placed in two different tubes, one containing K_2_-EDTA anticoagulant and the other without anticoagulant with the purpose of obtaining serum.

*Inclusion Criteria*. Patients diagnosed with *β*-thalassemia minor or iron deficiency anemia and normal subjects that needed to be older than 1 year old were included.

*Exclusion Criteria*. People under vitamin intake, people who smoke, with diabetes, coronary heart disease, rheumatoid arthritis, dyslipidemia, hypertension, malignancy, chronic liver disease, and renal dysfunction, and patients under iron therapy during 21 days prior to analysis or who have received transfusions in the last three months were excluded.

*Hematological Studies*. Total blood count was performed in hematology analyzer Sysmex KX-21N (Kobe, Japan). The diagnosis of the *β*-thalassemia trait was performed by cellulose acetate hemoglobin (Hb) electrophoresis at alkaline pH and HbA_2_ quantification by microcolumn chromatography (BioSystems, Barcelona, Spain). IDA diagnosis was made by determining serum iron, total iron binding capacity (TIBC), and transferrin saturation (SAT) through colorimetric method (Wiener Lab, Rosario, Argentina). SAT lower than 16% was considered diagnostic of IDA.

*Erythrocyte Catalase Activity (ECAT)*. The enzyme was analyzed in K_2_-EDTA anticoagulated whole blood using Góth technique [22]. Absorbance (*A*) of the yellow complex of molybdate and hydrogen peroxide was measured at 405 nm against blank 3. Sample contained 1,0 mL substrate (65 *μ*mol/mL H_2_O_2_ in 60 mmol/L sodium-potassium buffer pH 7,4) and 30 *μ*L hemolysate, and after 60 seconds the enzymatic reaction was stopped by adding 1,0 mL of 32,4 mmol/L ammonium molybdate. Blank 1 contained 1,0 mL substrate, 1,0 mL molybdate, and 30 *μ*L hemolysate; blank 2 contained 1,0 mL substrate, 1,0 mL molybdate, and 30 *μ*L buffer; blank 3 contained 1,0 mL buffer, 1.0 mL molybdate, and 30 *μ*L buffer. One unit of catalase decomposes 1 *μ*mol of H_2_O_2_ in 1 minute under these conditions and it is related to 1 L of whole blood. Catalase activity was expressed in Mega Units/L (MU/L) and was calculated with the following formula:

(1) Erythrocyte catalase activity MU/L=A blank  1−A sample×4,26×100A blank  2−A blank  3.

*Molecular Analysis*. Characterization of *β*-thalassemic mutations was realized by real-time PCR. Genomic DNA isolation was performed with High Pure PCR Template Preparation Kit (Roche Diagnostics). PCR, dissociation curves, and subsequent analysis were executed on the LightCycler 2.0 (Roche) equipment, simultaneously measuring signals from two different fluorophores. Primers were designed to amplify a 587 bp region of *β*-globin gene: Forward Primer, 5′-gctgtcatc act acctca tag-3′; Reverse Primer, 5′-gct gcaagtcaccactca g-3′. Two combinations of hybridization probes labeled with different fluorophores were used [23].

*Statistical Analysis*. Results were analyzed using SPSS 21.0 statistical program. The results were reported as Media ± Standard Deviation. For comparison Student *t*-Test and ANOVA were used. A significance level of *p* < 0.05 was adopted. The influence of iron serum on catalase values, independent of the group (normal, IDA, or BTT), was evaluated by simple regression analysis.

## 3. Results

Ninety-eight individuals were studied of which 67 were normal (N group), 21 with *β*-thalassemia trait (BTT group), and 10 with iron deficiency anemia (IDA group).  Table 1 shows the results for catalase activity, hematological parameters, and iron status in all BTT and IDA patients. Subjects were divided according to age: children (≤12 years), adolescents (13–18 years), adults (19–59 years), and older adults (≥60 years). ANOVA detected no significant differences (*p* = 0,187) in ECAT between the stated groups.

Five individuals, who were more than 60 years old, were normal (5/67, 7.5%), 2 *β*-thalassemia carriers (2/21, 9.5%), and 2 IDA (2/10, 20%). Only one of these subjects, which belonged to the normal group, showed increased catalase activity (139 MU/L). Apparently, age has no influence in the ECAT of this population.

In order to establish the normal range of catalase activity, 5th and 95th percentiles of N group results were determined. A range from 70,0 to 130,0 MU/L was obtained.

Increased catalase activity in 14% (3/21) of BTT subjects was observed. No significant differences (*p* = 0,245) were observed when N and BTT groups were compared.

**Figure 1:**
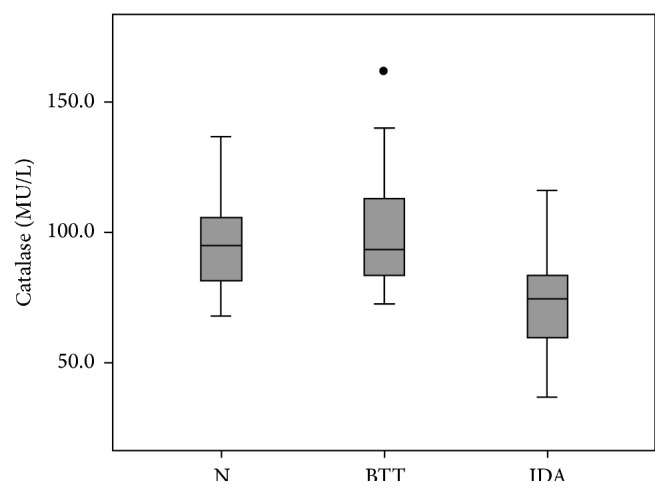
Erythrocyte catalase activity in *β*-thalassemia minor, iron deficiency anemia, and normal subjects. *p* < 0,05 between IDA and N groups. N, normal; IDA, iron deficiency anemia; BTT, *β*-thalassemia trait.

Four IDA individuals (40%) showed decreased catalase activity. No significant differences between men and women were detected in all groups. When N and IDA groups were compared, significant difference (*p* = 0,000) was observed, since the IDA subjects had lower values than N group (Figure 1).

Table 2 shows the results in N, BTT, and IDA groups according to sex. Female IDA subjects have significant differences (*p* < 0,05) with normal group in all parameters, except TIBC. Catalase in male IDA patients did not demonstrate significant differences (*p* > 0,05) when compared with normal and BTT groups.

The *β*-thalassemia mutations detected in order of frequency were codon 39 (C→T) (5 subjects), IVS-I-110 (G→A) (5 subjects), IVS-I-1 (G→A) (4 subjects), and IVS-I-6 (T→C) (2 subjects), and in 5 cases the mutation could not be assigned. The small number of patients for each group prevented the comparative study between them. Therefore differences between *β* ^0^ (9 subjects; ECAT = 104,6 ± 31,6 MU/L) and *β* ^+^ (7 subjects; ECAT = 91,8 ± 17,4 MU/L) groups were studied, which were not significant (*p* = 0,359).

There was no influence of iron levels on catalase values (*r* ^2^ = 0,153) in these samples.

## 4. Discussion

Oxidative stress is defined as the interruption of balance between oxidants and reductants within the body, due to the excess production of peroxides and free radicals. During the course of metabolism, superoxide anion is converted to H_2_O_2_ by ubiquitous enzyme SOD. Normally H_2_O_2_ is converted to innocuous compounds by the action of catalase and peroxidase. But if free iron is available, it reacts with H_2_O_2_ to form hydroxyl radicals which are extremely reactive species leading to depolymerisation of polysaccharide, DNA strand breakage, inactivation of functional proteins, and other events [24]. Therefore, this imbalance will cause damage to cellular components and tissues in the body leading to OxS, and catalase has a role in it.

Kósa et al. [25] revealed a significant decrease in catalase activity of 43 *β*-thalassemia carriers and attributed it to catalase protein damage by increased free radicals and H_2_O_2_. Another study reported increased levels of antioxidant enzymes like SOD, catalase, and GPx in RBCs of *β*-thalassemia minor individuals and near normal values of these enzymes in RBCs of *β*-thalassemia major patients [26]. They concluded that *β*-thalassemia minor RBCs react to increased OxS rising activities of antioxidant enzymes, while in *β*-thalassemia major normal antioxidant enzyme levels are due to the presence of normal RBCs from multiple blood transfusions. However, Boudrahem-Addour et al. [27] observed a significant increase (*p* < 0,05) of catalase activity in *β*-thalassemia major and intermedia. The present results, in concordance with Gerli et al. [26], showed that some BTT subjects had increased catalase activity.

Several authors reported increased antioxidant capacity in BTT individuals, but they do not measured catalase activity [28, 29]. Instead, they used a novel automated measurement method and Trolox equivalent antioxidant capacity.

In the catalase activity comparison between *β* ^0^ and *β* ^+^ thalassemia traits, the results were not significant. Also, Kósa et al. [25] did not find significant differences and concluded that catalase activity was not related to specific *β*-thalassemia mutations. Furthermore, Labib et al. [30] reported no differences in total antioxidant capacity of *β* ^0^ and *β* ^+^ thalassemia traits.

The normal range of erythrocyte catalase activity was slightly lower than the one reported by another authors [31], which was 80,3 to 146,3 MU/L. Difference may be due to the racial characteristics of the population, methodological differences, and because they used 2,5th and 97,5th percentiles to establish normal range. Men and women have no significant differences in what catalase activity concerns. Instead, Vitai and Góth [31] found slightly higher values in male subjects.

In this study, like Ondei et al. [29], there was no relationship between ECAT and iron serum. In *β*-thalassemia major and intermedia, iron excess can lead to organ damage, especially in liver and heart, and to endocrine dysfunction [32]. Recent studies have shown the importance of other markers such as non-transferrin bound iron (NTBI) and labile plasma iron (LPI) to detect iron excess in thalassemia patients due to the direct correlation of these markers with the formation of free radicals [33].

Decreasing serum iron concentration causes insufficient Hb synthesis with subsequent reduction of erythrocytes proliferation. Iron deficiency also affects the production of other iron-containing proteins, such as cytochrome, myoglobin, catalase, and peroxidase. Therefore, a decreased catalase activity could be anticipated in iron deficiency and has been corroborated in this study, in agreement with other works [21, 34, 35]. However, Madhikarmi and Murthy [20] detected an unexplained increase of catalase activity in IDA, and Tekin et al. [36] reported no differences in SOD and catalase activities between IDA patients and controls. Bay et al. [37] compared antioxidant capacity between IDA and normal subjects and detected no significant differences.

Increased OxS have been reported in patients with IDA [3, 21, 35], which occurs primarily in RBC membrane. ROS membrane surface contributes to deformability alteration [38] and phosphatidylserine exposure, which have been used to explain the reduction of RBCs life in IDA [39]. Also, in BTT an altered oxidative state was reported by some investigators [28, 30]. Therefore, OxS is present in the most common hypochromic microcytic anemia, IDA and BTT, being one of the determining factors of altered catalase activity. Hypochromic microcytic RBCs of BTT and IDA individuals have been studied in the past decades and OxS contribution in reducing the RBCs useful life has been documented. Previous studies on the catalase activity of hypochromic microcytic anemia such as IDA and BTT have reported disagreeing results [20, 21, 25, 26, 34, 35]. In the present work, catalase activity was decreased in IDA and increased in some BTT subjects, with no significant differences between beta-thalassemia mutations. These results will help to clarify how the catalase activity works in these anemia types. The low number of samples is a limitation of this report. Probably, the incorporation of a larger number of participants will allow revealing hidden differences in current work.

## Figures and Tables

**Table 1:** Laboratory data of *β*-thalassemia trait and iron deficiency anemia patients.

Groups	Age [years]	HTO [L/L]	HB [g/L]	ECAT [MU/L]	Fe [*μ*g/dL]	TIBC [*μ*g/dL]	SAT [%]
IDA group							
Female (6)							
P1	3	0,26	64	116,2	13	262	5
P2	14	0,34	103	54,3	25	444	6
P3	76	0,37	118	61,0	33	293	11
P4	44	0,35	101	59,8	29	313	9
P5	43	0,38	117	81,3	43	358	12
P6	15	0,17	38	37,0	31	397	8
Male (4)							
P1	1	0,33	97	90,4	15	352	4
P2	1	0,31	89	83,4	18	323	6
P3	11	0,31	79	71,7	25	216	12
P4	63	0,40	115	77,0	32	403	8

BTT group							
Female (15)							
P1	12	0,35	106	95,3	78	297	26
P2	10	0,33	100	87,3	75	380	20
P3	57	0,37	107	113,9	34	233	15
P4	48	0,39	121	88,8	80	247	32
P5	33	0,37	108	87,9	90	285	31
P6	33	0,36	103	83,5	63	203	31
P7	34	0,34	104	83,6	50	180	28
P8	38	0,38	115	106,2	176	341	52
P9	63	0,38	110	73,0	81	238	34
P10	56	0,36	106	79,8	100	220	45
P11	11	0,35	105	75,2	103	322	32
P12	32	0,37	111	127,9	80	228	35
P13	22	0,35	104	86,5	104	332	31
P14	28	0,37	114	162,2	71	258	28
P15	26	0,34	101	140,1	83	283	29
Male (6)							
P1	45	0,39	115	135,4	259	266	97
P2	1	0,32	93	74,4	29	347	8
P3	12	0,38	112	93,4	66	244	27
P4	65	0,47	142	107,3	144	318	45
P5	21	0,40	121	113,3	127	208	61
P6	16	0,41	126	100,0	58	247	23

HTO, hematocrit; HB, hemoglobin; ECAT, erythrocyte catalase activity; Fe, serum iron; TIBC, total iron binding capacity; SAT, transferrin saturation; IDA, iron deficiency anemia; BTT, *β*-thalassemia trait; P, patient.

**Table 2:** Hematological parameters, iron status, and catalase according to pathology and sex (Media ± Standard Deviation).

Pathology Sex	Age [years]	HTO [L/L]	HB [g/L]	ECAT [MU/L]	Fe [*μ*g/dL]	TIBC [*μ*g/dL]	SAT [%]
IDA group							
Female (6)	20 ± 19	0,31 ± 0,08^*∗*‡^	90 ± 32^*∗*‡^	68,3 ± 27,4^*∗*‡^	29 ± 10^*∗*‡^	344 ± 68^‡^	8 ± 3^*∗*‡^
Male (4)	19 ± 30	0,34 ± 0,04^*∗*^	95 ± 15^*∗*^	80,6 ± 8,1	22 ± 8^*∗*^	324 ± 79	7 ± 3^*∗*^
BTT group							
Female (15)	34 ± 17	0,36 ± 0,02^†^	108 ± 6^†^	99,4 ± 25,9	84 ± 32	270 ± 56	31 ± 9
Male (6)	27 ± 6	0,39 ± 0,05^†^	118 ± 16^†^	104,0 ± 20,4	114 ± 83	272 ± 52	44 ± 32
N group							
Female (39)	36 ± 13	0,41 ± 0,02	137 ± 10	96,8 ± 17,5	81 ± 18	301 ± 54	28 ± 11
Male (28)	37 ± 15	0,44 ± 0,02	147 ± 9	93,1 ± 15,4	94 ± 28	282 ± 38	34 ± 11

^*∗*^ *p* < 0,05 between IDA and N groups by sex; ^†^ *p* < 0,05 between BTT and N groups by sex; ^‡^ *p* < 0,05 between IDA and BTT women.

IDA, iron deficiency anemia; BTT, *β*-thalassemia trait; N, normal; HTO, hematocrit; HB, hemoglobin; ECAT, erythrocyte catalase activity; Fe, iron; TIBC, total iron binding capacity; SAT, transferrin saturation.
